# T124. CLINICAL DIFFERENCES BETWEEN URBAN AND RURAL SCHIZOPHRENIA

**DOI:** 10.1093/schbul/sby016.400

**Published:** 2018-04-01

**Authors:** Amine Larnaout, Rahma Nefzi, Amina Aissa, Rouaa Trabelsi, Emira Khelifa, Hanen Ben Ammar, Zouhaier El Hechmi

**Affiliations:** Hospital Al Razi

## Abstract

**Background:**

Schizophrenia is a highly heritable disease; yet heritability rate could never exceed 80% in most studies. Genetic factors could never then, by their own, account for the whole risk for developing schizophrenia. Thus, environmental factors co-play a major role. Urbanity is now well established to be one of the most influential environmental factors for developing severe mental illnesses. A strong association between exposure to urban environment and the risk of developing schizophrenia, was demonstrated and several studies have shown that living in an urban environment increases the risk for developing schizophrenia.

The aim of this study was to examine the clinic biological characteristics of patients with schizophrenia according to their rural or urban condition.

**Methods:**

This study was based on 106 patients with a DSM 5 diagnosis of schizophrenia in period of remission. Sociodemographic and clinical characteristics were assessed. PANSS scale, Calgary depression scale, CGI scale were used to assess different features of the disease and biological variables (CBC, renal and liver markers, inflammatory markers: CRP and CRP hs) were evaluated. Two groups were formed according to the patient’s urban or rural birth and early living.

**Results:**

There were 71 urban and 35 rural patients. The mean age was 42,7 years in the urban group and 42,3 years in the rural group. Socio demographic characteristics were similar between the two groups. Patients with urban background had a higher score on the negative subscale (p=0,01) and the general psychopathology subscale (P=0,025) of the PANSS compared with rural patients. Otherwise, compared to patients with a rural birth and early living, urban patients showed more depressive symptoms on the Calgary depression scale (p=0,039). There were no significant differences between the two groups regarding the biological variables tested.

**Discussion:**

The association between urban upbringing and risk for developing schizophrenia is well established. Nevertheless, relationship between epidemiological factors and different symptom dimensions is still poorly understood. Our study found a significant association between patients’ urban background and negative as well as disorganization and depressive symptoms. Most of published studies found a correlation between urban upbringing and positive psychotic symptoms in schizophrenia. To our knowledge, no similar result has ever been found before. Otherwise, there were several studies showing an association between disorganization and depressive symptoms with urban upbringing of the patients.

